# Broccoli: A Multi-Faceted Vegetable for Health: An In-Depth Review of Its Nutritional Attributes, Antimicrobial Abilities, and Anti-inflammatory Properties

**DOI:** 10.3390/antibiotics12071157

**Published:** 2023-07-07

**Authors:** Rahamat Unissa Syed, Sivakumar Sivagurunathan Moni, Mohammed Khaled Bin Break, Weam M. A. Khojali, Mohammed Jafar, Maali D. Alshammari, Karim Abdelsalam, Soha Taymour, Khetam Saad Mutni Alreshidi, Manal Mohamed Elhassan Taha, Syam Mohan

**Affiliations:** 1 Department of Pharmaceutics, College of Pharmacy, University of Hail, Hail 81442, Saudi Arabia; 2 Medical and Diagnostic Research Centre, University of Hail, Hail 55473, Saudi Arabia; m.binbreak@uoh.edu.sa (M.K.B.B.); we.ali@yo.edu.sa (W.M.A.K.); 3 Department of Pharmaceutics, College of Pharmacy, Jazan University, Jazan 45142, Saudi Arabia; drsmsivakumar@gmail.com; 4 Department of Pharmaceutical Chemistry, College of Pharmacy, University of Hail, Hail 81442, Saudi Arabia; maali.alshammari@uoh.edu.sa; 5 Department of Pharmaceutical Chemistry, Faculty of Pharmacy, Omdurman Islamic University, Al Khartoum 14415, Sudan; 6 Department of Pharmaceutics, College of Clinical Pharmacy, Imam Abdulrahman Bin Faisal University, P.O. Box 1982, Dammam 34212, Saudi Arabia; mjomar@iau.edu.sa; 7 Department of Clinical Pharmacy, College of Pharmacy, Jazan University, Jazan 45142, Saudi Arabia; drkarimpharmd@gmail.com (K.A.); sohataymour@yahoo.com (S.T.); 8 College of Pharmacy, University of Hail, Hail 81442, Saudi Arabia; khetam.albarrak@gmail.com; 9 Substance Abuse and Toxicology Research Centre, Jazan University, Jazan 45142, Saudi Arabia; mtaha@jazanu.edu.sa (M.M.E.T.); smohan@jazanu.edu.sa (S.M.); 10 Center for Transdisciplinary Research, Department of Pharmacology, Saveetha Dental College, Saveetha Institute of Medical and Technical Science, Saveetha University, Chennai 602105, India; 11 School of Health Sciences, University of Petroleum and Energy Studies, Dehradun 248007, India

**Keywords:** broccoli, cruciferous vegetable, nutritional powerhouse, medicinal properties, antibacterial, antioxidant, anti-inflammatory, anti-cancer

## Abstract

Broccoli, *Brassica oleracea* var. italica, has recently gained considerable attention due to its remarkable nutritional composition and numerous health benefits. In this review, the nutritional aspects of broccoli are examined, highlighting its rich nutrient content and essential bioactive compounds. The cruciferous vegetable broccoli is a rich source of several important nutrients, including fiber, vitamins (A, C, and K), minerals (calcium, potassium, and iron), and antioxidants. It has also been shown to contain bioactive compounds such as glucosinolates, sulforaphane, and indole-3-carbinol, all of which have been shown to have significant health-promoting effects. These chemicals are known to have potent antioxidant, anti-inflammatory, and anticancer effects. This review article aims to comprehensively examine the diverse spectrum of nutrients contained in broccoli and explore its medicinal potential to promote human health.

## 1. Introduction 

**Figure 1:**
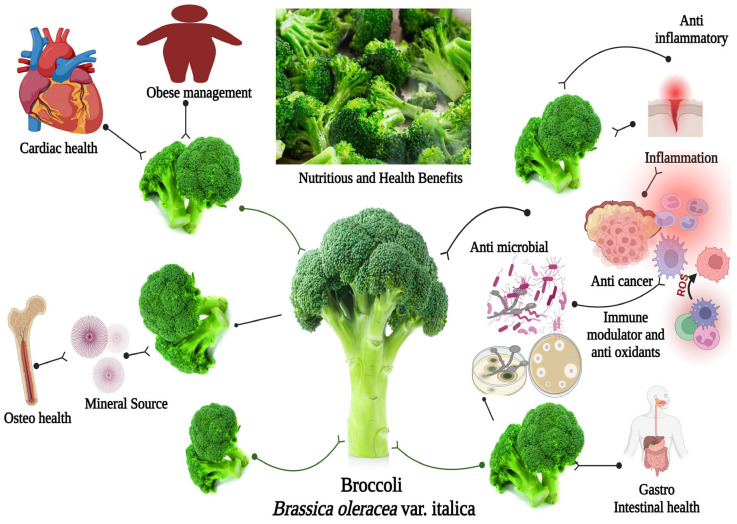
Nutritional and health benefits of broccoli. This figure was created with BioRender.com (accessed on 1 May 2023), Bio Render, Canada.

The prevention of diseases has always been considered better than the cure by various therapeutic measures. Health and nutrition are intimately related. Our bodies depend on the nutrients, energy, and chemicals in food to grow properly, develop, and stay healthy. A healthy diet is essential for preserving good health and reducing the risk of many diseases. Vegetables are an essential part of a balanced diet and play an important role in promoting good health. Despite the fact that vegetables have many health benefits. The dietary recommendations by health and nutrition experts have been repeatedly emphasizing the importance of the consumption of fruits and vegetables. Therefore, the consumption of selected vegetables and fruits to prevent certain diseases is of great importance to human society. Broccoli (*Brassica oleracea* L. var. italica) belongs to the Brassicaceae family and has more divided and stalked leaves. The main head consists of clusters of fully differentiated flower buds arranged less densely on longer stems. Sprouting forms of broccoli bear many small flower heads. It grows as an annual herb reaching 400 mm in the vegetative phase and 1–2 m at the end of the flowering period [1,2]. The pharmaceutical importance of broccoli is widely known as antimicrobial, antioxidant, anticancer, immunomodulator, antidiabetic, hepatoprotective, cardioprotective, and anti-amnesic [3,4,5,6,7,8,9]. Over the past five decades, socioeconomic development in Saudi Arabia has improved. Development has improved in the areas of basic education, health, environmental factors, urban migration, and lifestyle modifications, with a decrease in the incidence of communicable diseases but an increase in the incidence of non-communicable diseases, possibly due to the stressful life in this highly modern society. Researchers have recently reported that many non-communicable diseases have increased dramatically due to dietary factors. Cruciferous vegetables are high in fiber, low in calories, and rich in vitamins and minerals, which are beneficial for normal human physiological functions [4]. Dietary recommendations from health and nutrition experts have repeatedly emphasized the importance of consuming fruits and vegetables [10]. Many researchers reported that naturally occurring vegetables may be necessary for maintaining heart health. For example, the consumption of β-carotene and lycopene has been associated with a lower risk of heart disease [11]. Similarly, the daily consumption of broccoli may reduce the development of many diseases. Undoubtedly, the development of innovative anti-inflammatory drugs with antibacterial properties is one of the most advanced areas of medical research. These synergistic therapies are designed to simultaneously have anti-inflammatory and antibacterial effects, potentially helping to treat a wide range of inflammatory and infectious diseases. A single drug molecule that has both anti-inflammatory and antibacterial properties can potentially treat multiple features of diseases. For example, infections can trigger an inflammatory response in the body. Broccoli (*Brassica oleracea* var. italica) is a popular vegetable in culinary preparations and has great pharmaceutical importance due to its multiple applications (Figure 1). This review article explores the nutritional and pharmaceutical potentials of broccoli, focusing on the synergistic effects of anti-inflammatory and antibacterial potentiality for a better understanding.

## 2. Pharmaceutical Importance of Broccoli

### 2.1. Nutritional Source

Broccoli is often considered a nutritional powerhouse because of its numerous health benefits and nutrient density (Table 1). Broccoli is an excellent source of vitamins C, K, and A. It also contains several important minerals, such as potassium, calcium, and iron. Broccoli contains several antioxidants, including vitamins C and E, β-carotene, and various flavonoids [12,13]. Antioxidants help protect cells from damage caused by harmful free radicals and reduce the risk of chronic diseases [14]. Broccoli is a good source of dietary fiber, which aids digestion, promotes a feeling of fullness, and contributes to a healthy digestive system [13]. Broccoli’s fiber, antioxidants, and anti-inflammatory properties contribute to heart health. It can help lower cholesterol, maintain healthy blood pressure, and improve cardiovascular function. The high levels of vitamin A and other antioxidants in broccoli support eye health and may help prevent age-related macular degeneration and cataracts [14,15]. Its high vitamin C content boosts the immune system and promotes collagen production, wound healing, and iron absorption [15,16,17,18]. Broccoli is a good calcium source, essential for maintaining strong bones and preventing osteoporosis [19,20]. It also contains vitamin K, which is essential for bone health. Broccoli is low in calories but high in fiber, making it a filling food that can help control weight and promote a healthy metabolism [21]. The fiber content in broccoli supports a healthy digestive system, regulating bowel movements and promoting a healthy gut microbiome [22]. Overall, broccoli offers various health benefits due to its rich nutrient content. From promoting heart health to supporting digestion and bone health, this cruciferous vegetable provides a versatile and tasty way to improve your overall well-being.

### 2.2. Anti-Inflammatory, Antioxidant, and Anticancer Potential of Broccoli

Inflammation is a natural response of the immune system to protect the body from injury, infection, or other harmful stimuli. However, chronic inflammation can be detrimental to health and contribute to various diseases such as heart disease, arthritis, and certain types of cancer. Herbs and vegetables can positively reduce inflammation due to their high content of phytochemicals, antioxidants, and other bioactive compounds. Research suggests that sulforaphane, found in broccoli [21,23], may help reduce inflammation by inhibiting the activity of certain enzymes that promote inflammation in the body (Table 2). It has also been found to stimulate the production of antioxidant enzymes that protect cells from inflammation-related damage [24,25]. When inflammation persists over a long period, it can create an environment that promotes the growth and survival of cancer cells. Chronic inflammation can lead to the release of additional cytokines and growth factors that can stimulate cell proliferation and support the formation of new blood vessels to supply nutrients to the growing tumor. Inflammatory cells can also produce enzymes that degrade the extracellular matrix, allowing cancer cells to invade surrounding tissues and metastasize to distant organs. Several types of cancer are closely related to chronic inflammation [25,26]. For example, prolonged inflammation of the gastrointestinal tract, as occurring in inflammatory bowel disease (e.g., Crohn’s disease, ulcerative colitis), increases the risk of developing colorectal cancer. In addition, chronic hepatitis B or C virus infections can lead to liver inflammation and increase the likelihood of liver cancer (hepatocellular carcinoma). Similarly, chronic human papillomavirus (HPV) infections can cause cervix inflammation and contribute to the development of cervical cancer.

It is important to note that not all cancers are directly related to inflammation and that inflammation alone is insufficient to cause cancer. Genetic and environmental factors also play an essential role in cancer development. However, controlling chronic inflammation can help reduce the risk of certain cancers and improve outcomes for individuals diagnosed with cancer. Antioxidants and anti-inflammatory compounds are closely connected and often work together to promote health and protect the body against various diseases. Antioxidants help prevent or slow down oxidative damage caused by free radicals in the body. Free radicals are highly reactive molecules that can damage cells and contribute to various chronic diseases, including inflammation [28,29,30,31,32,33]. Antioxidants neutralize free radicals by donating an electron, stabilizing them, and preventing them from causing further damage. Broccoli is well known for its high antioxidant potential. Antioxidants help protect cells from damage caused by free radicals, unstable molecules that can lead to oxidative stress and contribute to various diseases [33]. Research has shown that oxidative stress, resulting from an imbalance between the production of free radicals and the body’s antioxidant defenses, can trigger and sustain chronic inflammation.

Reactive oxygen species (ROS) are chemically reactive molecules containing oxygen that are generated as byproducts of normal cellular metabolism. They are produced during various physiological processes in the body, including aerobic respiration, inflammation, and immune response. Examples of ROS include superoxide anions (O^2−^), hydrogen peroxide (H_2_O_2_), hydroxyl radicals (OH·), and singlet oxygen (^1^O_2_). ROS play a dual role in biological systems. On the one hand, they are important signaling molecules involved in cell growth, proliferation, and immune function. They participate in cellular signaling pathways and are necessary for normal physiological processes. On the other hand, ROS can also damage cells and tissues when their production exceeds the body’s ability to neutralize or detoxify them and are called oxidative stress [29,30,31]. Inflammatory cells produce ROS during the inflammatory response, which can further increase oxidative stress. They are naturally produced in the body during various cellular processes, including energy production. However, excessive production of ROS can lead to oxidative stress and DNA damage, potentially contributing to the development of cancer [32].

The inflammation process and ROS are interdependent processes that may influence each other. Production of ROS is a normal component of the inflammatory response; however, excessive ROS production or weakened antioxidant defenses can lead to oxidative stress, which perpetuates inflammation and contributes to a variety of diseases. Maintaining a balance between inflammation and reactive oxygen species is essential for optimal physiological function and overall health [33].

Antioxidants help counteract this oxidative stress by neutralizing, thereby reducing the potential DNA damage that could lead to cancer development. Antioxidants play a critical role in protecting cells and tissues from oxidative damage. This cellular protection can also prevent mutations and abnormalities that can lead to cancer. By neutralizing free radicals and reducing oxidative stress, antioxidants help maintain cellular integrity and promote healthy cellular function, which may reduce the risk of cancer development. Antioxidants and anti-inflammatory compounds are interconnected in their actions within the body [34]. Antioxidants help reduce oxidative stress, which can contribute to chronic inflammation. By reducing inflammation, antioxidants can help protect against various diseases. Chronic inflammation is closely linked to the development of cancer.

Cancer is the major threat to human society that causes death globally irrespective of socioeconomic context. Cancer is the leading cause of death worldwide, accounting for nearly 10 million deaths in 2020 [32]. The knowledge about the health benefits of vegetables and fruits is sprouting awareness among the public day by day. On the other hand, the incidence of cancer is also increasing everyday due to various reasons. Broccoli is a cruciferous vegetable that has gained significant attention in the field of cancer research due to its potential anti-cancer properties. It contains various bioactive compounds that have been shown to have beneficial effects on human health, including anti-cancer properties. 

Glucosinolates are a group of sulfur-containing compounds found in cruciferous vegetables such as broccoli, cauliflower, kale, Brussels sprouts, and cabbage. These compounds are responsible for the characteristic pungent aroma and bitter taste of these vegetables. Glucosinolates are secondary metabolites that serve as natural defenses in plants. When plant tissue is damaged by chopping or chewing, an enzyme called myrosinase encounters glucosinolates, leading to their degradation and the formation of various bioactive compounds. One of the major degradation products of glucosinolates is an isothiocyanate called sulforaphane, which has attracted considerable attention because of its potential health benefits. The isothiocyanate sulforaphane was first recognized as an enzyme inducer in the II phase and was associated with anti-cancer effects. Studies have shown that sulforaphane has a direct effect on cancer cell proliferation [34,35,36,37]. In addition, sulforaphane also shows various biological activities such as antihypertensive, cardioprotective, and complementary treatment in type 2 diabetes. Conversely, nitrile has no significant cancer-preventive effect [37,38,39,40,41,42]. 

Broccoli is rich in sulforaphane, a sulfur-containing compound that has been extensively studied for its anti-cancer properties. Sulforaphane has been found to have the ability to inhibit the growth of cancer cells and induce apoptosis in various types of cancer, including breast, prostate, lung, and colorectal cancers [43]. An earlier study revealed that sulforaphane enhances the drug-mediated cytotoxicity in SCC12 and SCC38 squamous cell carcinomas of the head and neck [44,45]. It works by modulating multiple cellular pathways involved in cancer development and progression in several types of cancer, including breast, prostate, lung, colon, and liver cancer. Recently, Zhang et al. (2022) reported that sulforaphane interferes with the RAF/MEK/ERK signaling pathway to inhibit actin stress fiber formation and thereby prevent breast cancer cell metastasis [46]. The preceding review suggests that sulforaphane exerts its therapeutic effects through a variety of mechanisms, such as detoxification of carcinogens and oxidants by blocking phase I metabolic enzymes and arresting the cell cycle in phases G2/M and G1 to inhibit cell proliferation. However, the most striking observation was the ability of sulforaphane to enhance the effects of several classes of anticancer agents, including paclitaxel, docetaxel, and gemcitabine, through additive and synergistic effects [46].

Indole-3-carbinol (I3C) is a compound found naturally in cruciferous vegetables such as broccoli, cauliflower, cabbage, and Brussels sprouts. It has been of interest to researchers due to its potential health benefits, particularly in relation to cancer prevention. Studies suggest that I3C may have anticancer properties. On the other hand, indole-3-carbinol is a well-known chemo preventive drug with a variety of biological effects, which include promoting tumor cell death and inhibiting angiogenesis and inflammation [47]. It is believed to exert its effects through various mechanisms, including altering estrogen metabolism, inducing cell cycle arrest, promoting apoptosis, and inhibiting angiogenesis. I3C has been described as a potent inducer of cytochrome-P450-dependent metabolism of estrogen. Estrogen is critical for the development of recurrent respiratory papillomatosis by promoting epithelial proliferation and enhancing human papillomavirus gene expression [48]. 

Quercetin is a flavonoid found in broccoli that has anti-inflammatory properties. It can inhibit the production of inflammatory substances and help reduce inflammation in the body. The MAPK pathway plays a role in cell signaling and the regulation of various cellular processes, including inflammation, and there is limited evidence specifically linking quercetin’s effect on this pathway. Quercetin has been shown to modulate several signaling pathways, including NF-κB and PI3K/Akt, which are involved in inflammation. However, the direct inhibition of the MAPK pathway by quercetin is not well established [34]. 

Broccoli is an excellent source of vitamin C, which is a potent antioxidant. Vitamin C scavenges free radicals and helps regenerate other antioxidants in the body, such as vitamin E [49,50,51,52,53]. It plays a crucial role in protecting cells and tissues from oxidative damage. Broccoli contains various flavonoids and phenolic compounds known for their antioxidant properties [52,53]. These compounds, such as kaempferol and quercetin, can neutralize free radicals and reduce oxidative stress in the body. Glucosinolates are sulfur containing plant secondary metabolites found in broccoli that are involved in cancer prevention and have antioxidant properties. Glucosinolates are converted into sulforaphane, has been shown to enhance the body’s natural antioxidant defenses and reduce oxidative stress [54,55,56]. Broccoli contains carotenoids such as β-carotene and lutein, which act as antioxidants. These compounds can help protect cells from damage caused by free radicals, particularly in tissues like the eyes and skin [56,57,58,59]. Broccoli is a good source of selenium, a mineral that is an essential component of antioxidant enzymes, including glutathione peroxidase [59,60]. These enzymes help neutralize harmful free radicals and reduce oxidative stress. Consuming broccoli and other antioxidant-rich foods as part of a balanced diet may help reduce oxidative stress, support cellular health, and lower the risk of chronic diseases associated with oxidative damage, such as cardiovascular diseases, certain cancers, and neurodegenerative disorders [61,62].

### 2.3. Antibacterial Properties

Antioxidants may enhance the efficacy of antimicrobial therapies. Some studies suggest that combining antioxidants with antimicrobial agents such as antibiotics or antiviral drugs may enhance their efficacy by reducing oxidative-stress-induced host tissue damage and supporting the immune system’s response to infection. While antioxidants are primarily known for their ability to counteract oxidative stress, some specific antioxidants have also been found to have natural antimicrobial properties. For example, certain plant antioxidants, such as flavonoids and polyphenols, have been shown to have antimicrobial activity against a range of microorganisms, including bacteria, viruses, and fungi [63,64,65,66].

ROS plays a critical role in the immune response against bacterial infections. When the body recognizes bacteria, immune cells such as neutrophils and macrophages are activated to eliminate the invading pathogens [67]. In addition, immune cells generate ROS, such as superoxide anions (O^2−^), hydrogen peroxide (H_2_O_2_), and hydroxyl radicals (OH^−^), through a process called respiratory burst. The production of ROS is a rapid and effective defense mechanism aimed at killing bacteria or inhibiting their growth and can directly damage bacterial cells. ROS can react with various cellular components of bacteria, including lipids, proteins, and nucleic acids, resulting in oxidative damage and disruption of essential cellular processes [68]. This oxidative stress can lead to membrane damage, protein dysfunction, and DNA/RNA damage, ultimately resulting in bacterial death [69]. ROS also functions as a signaling molecule in immune cells. It can regulate immune cell activation, migration, and cytokine production, which is essential for an effective immune response against bacterial infections [70]. While ROS effectively kills bacteria, some bacterial pathogens have evolved mechanisms to counteract or neutralize [68,71]. Certain bacteria produce enzymes such as catalase and superoxide dismutase that help break down ROS and protect the bacteria from oxidative damage. In this way, the bacteria can bypass the immune response and survive in the host. ROS serves as an effective antimicrobial agent that directly damages bacterial cells and helps eliminate pathogens. However, some bacteria have developed strategies to counteract and can evade the immune response. Therefore, the balance between the production of ROS and antioxidant defense is critical for an effective immune response against bacterial infections [68]. Even though antioxidants are primarily recognized for their function in combating oxidative stress and their potential health benefits, it has been discovered that certain antioxidants also possess antibacterial properties. Broccoli is often highlighted for its potential antibacterial effects, which can contribute to overall health and the prevention of bacterial infections. Broccoli contains certain compounds such as glucosinolates and isothiocyanates that have been shown to have antibacterial activity. Sulforaphane may have antibacterial activity against *Helicobacter pylori*, a bacterium associated with gastric ulcers and gastrointestinal infections [68,69,70,71,72]. In addition to sulforaphane, other compounds in broccoli, such as indole-3-carbinol and phenolic compounds, have also shown some antimicrobial activity in laboratory studies. These compounds have shown inhibitory activity against certain strains of bacteria, including *Escherichia coli* and *Staphylococcus aureus* [72,73]. On the other hand, biofilms can contribute to bacterial persistence and resistance, making them more difficult to treat with antibiotics. By inhibiting biofilm formation, bacterial load is reduced. In this context, 3,3’-diindolylmethane (DIM), a bioactive component of broccoli, may act as an inhibitor of biofilm formation and cause a reduction in bacterial load [74]. Bacterial contamination in food is a common cause of foodborne illnesses. Broccoli’s antibacterial properties, particularly its ability to inhibit the growth of certain bacteria, can help reduce the risk of foodborne infections. It can potentially hinder the growth of pathogens such as *Escherichia coli*, *Salmonella* sp., and *Listeria monocytogenes*, which are commonly associated with foodborne outbreaks. Antibiotic resistance is a growing concern in healthcare. Certain bacteria have become resistant to commonly used antibiotics, making infections more difficult to treat. Broccoli’s antibacterial properties, particularly its ability to inhibit the growth of antibiotic-resistant strains, have garnered interest as a potential natural alternative or adjunct to traditional antibiotics. Further research is needed to fully understand the effectiveness and mechanisms of action in this context.

## 3. Conclusions

The effect of broccoli highlights its significant potential as a functional food due to its multiple health benefits. It is a nutrient-rich vegetable with important vitamins, minerals, fiber, and other bioactive compounds. These nutrients and phytochemicals support overall health and wellness, including cancer prevention and reduced inflammation. The article highlights the importance of broccoli in a balanced diet due to its anti-inflammatory, antioxidant, anti-cancer, and potentially antibacterial effects. Several findings support the anti-inflammatory, antioxidant, anti-cancer, and antibacterial effects of broccoli. Further studies are needed to explore optimal dosages, preparations, and potential synergistic effects with other foods or therapies to maximize the health benefits of broccoli.

## Figures and Tables

**Table 1:** Broccoli: nutritional and medicinal properties.

Characteristics	Properties	References
Rich in vitamins and minerals	Broccoli is a rich source of antioxidants, including vitamins C, K, and A. It also contains several important minerals, including potassium, calcium, and iron. These antioxidants help protect the body from oxidative stress and reduce inflammation.	[11,13]
High in fiber	Broccoli is an excellent source of dietary fiber, which not only aids digestion but also promotes satiety and helps maintain a healthy digestive system. Dietary fiber has been associated with reduced inflammation in the body.	[13]
Antioxidant properties	Broccoli is an excellent source of various antioxidants, such as vitamins C and E, β-carotene, and various flavonoids. It is known that antioxidants reduce the risk of developing chronic diseases, as they protect cells from damage caused by dangerous free radicals.	[13,14]
Anti-cancer properties	The cruciferous family, which includes broccoli, is known for its possible anti-cancer properties. It contains glucosinolates, which the body can convert into substances that fight cancer.	[13,16]
Heart health	Broccoli’s anti-inflammatory, antioxidant, and fiber-rich properties support heart health. It can help lower cholesterol, maintain proper blood pressure, and promote cardiovascular health.	[17]
Eye health	The high content of vitamin A and antioxidants in broccoli promotes eye health and may prevent age-related macular degeneration and cataracts.	[18]
Immune system support	The vitamin C content of broccoli strengthens the immune system and promotes collagen production, wound healing, and iron absorption. Compounds such as indole-3-carbinol and diindolylmethane, both found in broccoli, have been shown to influence the immune system. These substances help regulate the immune response and reduce excessive inflammation.	[19]
Bone health	Broccoli is an excellent source of calcium, which is essential for healthy bone growth and prevention of osteoporosis. It also contains vitamin K, which is crucial for maintaining healthy bones.	[20]
Weight management	Broccoli is relatively low in calories but high in fiber, making it a filling food that can help with weight control and promote a healthy metabolism.	[21]
Digestive health	The high fiber content of broccoli contributes to a healthy digestive system, ensures regular bowel movements, and promotes the growth of good microbes in the intestine.	[22]

**Table 2:** Anti-inflammatory properties of broccoli.

Bioactive Compounds	Properties	References
Sulforaphane	Sulforaphane is a ubiquitous sulfur-containing compound found in broccoli. It has been shown to provide significant health benefits. It has potent anti-inflammatory properties. Studies have shown that sulforaphane can inhibit the production of inflammatory substances and reduce inflammatory markers.	[23,24,25]
Sulforaphane, indole-3-carbinol, isothiocyanates, and flavonoids	Broccoli is rich in antioxidants, vitamins, and minerals that protect the body from oxidative stress and inflammation: promoting heart health, supporting digestion and bone health. The compounds sulforaphane, indole-3-carbinol, isothiocyanates, and flavonoids have anti-inflammatory properties and can help reduce the production of pro-inflammatory molecules such as cytokines and prostaglandins.	[26]
Sulforaphane	Sulforaphane has been shown to influence the activity of immune cells involved in inflammation, such as macrophages and lymphocytes. Its potential as a natural immunomodulatory molecule is highlighted by the fact that sulforaphane has the ability to influence the activity of immune cells involved in inflammation. It can regulate the production of pro-inflammatory cytokines, such as interleukin-6 (IL-6) and tumor necrosis factor-alpha (TNF-alpha), by immune cells, thereby reducing inflammation.	[27,28]
Quercetin	Quercetin is a potent antioxidant flavonoid found in broccoli. It is a plant pigment. Broccoli extract can modulate several signaling pathways involved in inflammation, such as nuclear factor kappa B (NF-κB) and mitogen-activated protein kinases (MAPKs). These signaling pathways play a critical role in the expression of genes involved in inflammation. By regulating these signaling pathways, broccoli extract contributes to the control of the inflammatory response.	[29]

## Data Availability

Not applicable.
